# Tobacco use in Haiti: findings from demographic and health survey

**DOI:** 10.1186/s12889-023-17409-6

**Published:** 2023-12-14

**Authors:** Bénédique Paul, David Jean Simon, Vénunyé Claude Kondo Tokpovi, Ann Kiragu, Ketty Balthazard-Accou, Evens Emmanuel

**Affiliations:** 1 Department of Agro-socio-economics, Chibas, Université Quisqueya, Port-au-Prince, Haiti; 2 Groupe d’Etude sur les Sciences de la Durabilité, Université Quisqueya, Port-au-Prince, Haiti; 3 Bureau d’Etudes et de Recherche en Statistiques Appliquées, Suivi et Evaluation (BERSA-SE), Port-au-Prince, Haiti; 4 Groupe de Recherche sur l’inadaptation psychosociale (GRIP), University of Laval, Québec, Canada; 5 Department of Law and Political and Social Sciences, University of Sorbonne Paris Nord, Paris, France; 6 Espace universitaire One Health, Université Quisqueya, Port-au-Prince, Haiti

**Keywords:** Tobacco use, Prevalence, Demographic and health survey, Consumption behaviors, Haiti

## Abstract

**Introduction:**

Although tobacco has harmful effects on the physical and mental health of individuals, its use remains significant, according to the World Health Organization. To understand this phenomenon, studies have been carried out in many countries around the world, while in Haiti where more than 5,000 people die each year due to tobacco use, little is known about the use of this substance. The aim of this study was to examine the prevalence and the factors associated with tobacco use in Haiti.

**Methods:**

We used data from the 2016/17 Haitian Demographic Health Survey. Both descriptive and multivariate analyses were conducted using STATA 16.0 software to assess the prevalence and identify factors associated with tobacco use. Results were reported as adjusted odds ratios with 95% confidence intervals. Statistical significance was declared at *p* < 0.05.

**Results:**

The prevalence of tobacco use was estimated at 9.8% (95% CI: 9.2–10.4) among men and 1.7% (95% CI: 1.5–1.9) among women. Although the prevalence of tobacco use was low among young people, it increased with age. Respondents aged 35 and above, with no formal education, non-Christians, divorced/separated/widowed, from poorest households, rural areas, “Aire Métropolitaine de Port-au-Prince” region, with high media exposure had a higher likelihood of tobacco use.

**Conclusion:**

The low prevalence of tobacco use among Haitian women and youth represents a public policy opportunity to prevent these vulnerable groups from starting smoking. Adult male smokers should also be targeted by appropriate policy to reduce the different health burdens associated with tobacco, both for the smokers and other people they may expose to passive smoking. Government and health sector stakeholders, along with community leaders, should create and enforce awareness strategies and rules to control advertisements that encourage irresponsible and health-risky consumption behaviors.

## Introduction

Tobacco use, smoked and smokeless, is a critical global health problem with devastating consequences and adverse health, social and economic effects [1, 2]. While the adoption of the World Health Organization’s (WHO) Framework Convention on Tobacco Control in 2003 aimed at reducing and eventually eliminating disability and death caused by tobacco use [3], approximately 1.3 billion people globally, still use tobacco of which 80% live in low- and middle -income countries [4]. Recent WHO estimates show that, globally tobacco-related diseases are responsible for 8 million deaths each year [4]. Although cigarette use is by far the dominant form of tobacco use worldwide with an estimated 1.14 billion smokers [1], smokeless tobacco use accounts to more than 300 million people with its trends showing a sharp rise in many parts of the world partly due to the misconception that it is a safer alternative to smoking [5]. In addition, while data suggest that smoking prevalence has decreased [6], smoking during pregnancy remains prevalent in many countries [1].

The detrimental effects of tobacco on physical health and well-being are widely documented. The consumption of tobacco is the leading risk factor for premature deaths, particularly among males [1]. Numerous studies have shown that cigarette smoking is the major cause of lung cancer [7–9], with the duration of smoking being the strongest determinant risk [10], and the risk increasing in proportion to the number of cigarettes smoked [11]. In addition, smoking is the most important cause of chronic pulmonary diseases [12, 13], the risk of dying from any respiratory disease being higher for smokers than nonsmokers [11]. Moreover, smokeless tobacco is associated with different types of cancers, in particular head and neck cancers [14, 15], and an increased risk of cardiovascular deaths [16, 17]. Furthermore, studies suggest that tobacco use, for both smoked and smokeless products, among women of reproductive age group, particularly during pregnancy, increases the risk of fetal growth restriction, preterm births, stillbirths, perinatal deaths, sudden infant death syndrome and placental abnormalities [18, 19] and is also to linked to low birth weight [20].

Several factors have been found to influence tobacco use. Studies show that a vast majority of tobacco use typically begins during adolescence [14, 21, 22], and with an increase of age, the likelihood of consuming tobacco products significantly increase [23, 24]. Furthermore, gender is associated with tobacco use with men smoking more than women [25, 26], mainly those in the lower socioeconomic category [27]. Studies in various sub-Saharan African countries and in Nepal have found that older men are more likely to smoke and smoke greater quantities than younger men [28–30]. But, studies show that tobacco consumption in females is mostly in form of smokeless tobacco [23]. In addition, education has been found to be an important determinant of tobacco use regardless of the type with uneducated males and females at higher risk of tobacco use [23, 31, 32]. Other factors influencing tobacco use include exposure to media [33] and religion with Muslims and Christian being less likely to consume tobacco products [27, 34].

In Haiti, studies are lacking regarding tobacco use, although it has been reported that more 5,000 people die each year due to tobacco smoking [35]. Existing studies have focused more on alcohol use among Haitian women living with HIV and their effects on sexual behavior of male adolescents [36]. To the best of the authors’ knowledge, no study has evaluated prevalence and determinants of tobacco use in Haiti. However such a study is crucial to better inform policy, particularly when considering the absence of enforced regulation regarding the sale of tobacco products, especially in places frequented by young people. This new information on Haiti should be an impetus for even more vigorous programs to reduce tobacco use particularly smoking. Therefore, the present study aimed to examine the prevalence and explore the individual and community-level factors associated with tobacco use in Haiti.

## Materials and methods

### Study setting

Haiti is divided into 10 geographical departments (“Ouest” containing Aire Métropolitaine de Port-au-Prince region and Reste-Ouest, “Sud”, “Sud-Est”, “Grand’Anse”, “Nippes”, “Nord”, “Nord-Ouest”, “Nord-Est”, “Centre”, and “Artibonite”) which are divided into 42 districts, 140 municipalities and 570 communal sections. It has a population of 11.7 million people (of which 50.5% are women) in a total land area of 27,750 km^2^ [37] with 58% of the population residing in urban areas [38]. Haiti has a very young population structure: the median age is estimated at 24 years and less than 5% of people is 65 years or older [37]. Economically, it is the Western Hemisphere’s less developed country with a GDP per capita of 1829.6 (current USD) [39]. Agriculture, including tobacco whose production has decreased considerably, is continuously declining. It contributed about 16% of Haiti’s GDP in 2022 [40], but it remains the most important source of income for many Haitian households in rural areas [41].

### Data source and sample

Data used in this study derived from the most recent Haitian Demographic and Health Survey (HDHS) conducted between November 2016 and April 2017 by the Haitian Institute for Children (HIC), with technical support from Haiti National Bureau of Statistics (HNBS), Haitian Ministry of Public Health (HMPH), ICF International and the United States Agency for International Development (USAID). The 2016/17 HDHS is a nationally representative survey that collected data on household population and characteristics including information on access to toilets, fertility, marriage, and sexual activity, nutrition, malaria, HIV-AIDS, maternal and child health, adult and childhood mortality, women’s empowerment, and domestic violence. It also provides information on the consumption of substances such as alcohol, tobacco, among others, and other health issues relevant to the achievement of the Sustainable Development Goals (SDGs). The survey participants in this large national survey were selected representatively from all the eleven (11) regions of the Republic of Haiti, stratified into rural and urban areas [42].

Data collection was done through multistage sampling design. The first stage comprised the selection of Enumeration Areas (clusters) from the national sampling frame (i.e. the 2003 Haiti population and housing census, which was partially updated in 2011 by HNBS), and household listing. This is followed by the second stage through listing of households within the selected clusters, providing sampling frame where the households were randomly selected from all the clusters to provide enough estimates for key indicators with acceptable precision. The 2016/17 HDHS generated data from 13,405 households from which 15,513 women, and 11,886 men aged 15–64 years were successfully interviewed. The detailed methodology of the survey design, sampling, and the data collection methods are described in the full report [42].

### Inclusion and exclusion criteria

All women and men questioned about tobacco use at the time of the survey constituted our study population, resulting in a total weighted sample of 24,166 participants (14,371 women and 9,795 men). Women and men who were not questioned about tobacco use were excluded from the study.

### Study variables

#### Dependent variable

The outcome variable was tobacco use, measured as a binary variable with the response categories as 1 “Yes” (If respondents reported using any type of tobacco every day or some days), and 0 “No” (if respondents reported not using tobacco at all). Tobacco use included cigarettes, pipe full of tobacco, chews tobacco, and nasal snuff tobacco.

#### Independent variables

The independent variables of this study were categorized as individual and community-level variables. The individual-level factors were: age (categorized as less than 25, 25–34, 35 and above), education level (no formal education, primary, secondary, and higher), religion (Christian and non-Christian), wealth index (poorest, poorer, middle, richer, and richest), working status (yes and no), and marital status (never in union, in union and divorced/separated/widowed). The community-level factors were: place of residence (urban and rural), region (Aire Métropolitaine de Port-au-Prince, Reste-Ouest, Grand Sud, Grand Nord, Artibonite/Centre, Grand’Anse/Nippes), and community level media exposure. The community level media exposure was created by combining the responses to the following three questions (a) frequency of reading newspaper or magazine, (b) frequency of listening to radio, (c) frequency of watching television, (d) frequency of using internet. First, we generated an individual-level indicator by differentiating participants who reported being exposed to all these mass media almost every day, as having “high exposure”, from those who did not as having “low exposure”. Second, we aggregated the scores of individuals at the community-level to derive the proportion of participants with high exposure for every community/cluster. Most individual and community-level explanatory variables were chosen based on similar factors considered in previous literatures on tobacco use as well as their availability in the data files [24, 43–47].

### Data management and analysis

STATA software version 16 (StataCorp, College Station, TX) was used for data recoding and analysis. Univariate descriptive statistics (frequencies, percentages, mean, and standard deviation) were performed to describe socio-demographic profiles of the respondents. Then, bivariate analyses were carried out to assess the prevalence of tobacco use by socio-demographic parameters, and to explore independent associations (Rao-Scott chi-square test) between the outcome variable and each covariate. Further, to identify significant factors associated with tobacco use, a multilevel binary logistic regression analysis was applied because the 2016/17 HDHS data has a hierarchical structure that violates the independent assumptions of the standard logistic regression model. In our study, four models were fitted: null model (Model-0), model 1 (Model-I), model 2 (Model-II), and model 3 (Model-III). The null model was fitted with only the outcome variable [48]. Model 1, model 2, and model 3 were fitted using individual-level variables, community-level variables, and both individual and community-level variables, respectively. Results of the multilevel binary logistic regression were reported as adjusted odds ratios (aOR) with their corresponding 95% confidence intervals (CIs). The random effect was interpreted using the Intra-class Correlation Coefficient (ICC) and the Proportional Change in Variance (PCV) and compared across the progressive models by looking at them. Additionally, to detect potential multicollinearity, we used the variance inflation factor (VIF) at a cut-off point of 10 [49–51]. None of the variables displayed multicollinearity problems (all VIF < 10; Mean VIF < 2.5). Log-likelihood and Akaike Information Criterion (AIC) were used to verify model fitness, and a model with the highest log-likelihood and lowest AIC has been deemed as a best-fit model [52]. Sample weight was used to compensate for the unequal probability of selection between the strata that were geographically defined and the “svyset” command was applied to enable the corrections for clusters, stratification, and sample weights [53]. Statistical significance was set at *p* < 0.05. To avoid biased results, the women and men datasets have not been merged [54]. The study considered each dataset separately.

### Ethics

As stated earlier, this study is based on a secondary analysis of publicly available data, under free registration and request (https://dhsprogram.com/data/available-datasets.cfm). The 2016/17 dataset used was obtained from DHS Program with official permission as of May 3, 2022. The HDHS survey protocol obtained ethical clearance from the National Ethics Committee of Haiti and the Institutional Review Board of ICF/USAID. During the data collection, informed consent was obtained by participants and/or their legal guardians (under 16 years of age). Since this is a study involving secondary database, we were waived the need for additional informed consent. The participants’ anonymity and confidentiality were assured. All methods were carried out in accordance with relevant guidelines and regulations.

## Results

### Socio-demographic profiles of the study participants

Background characteristics of the study population are shown in Table 1. About 30% of the women were 35 and above, and 41.8% were under 25 years old. The mean age of the women was 28.5 (SD ± 9.8). More than 90% of them were Christians, 53.2% were from rural areas, 25.3% came from the “Aire Métropolitaine de Port-au-Prince” region, 15.9% in “Reste-Ouest”, 19.3% in “Grand Nord”, and 20.9% in “Artibonite/Centre”. Almost half of women (49.2%) had secondary education level, 7.3% had higher education level, and 13.3% had no formal education. Nearly one-third (32%) were in the poor (poorest and poorer) wealth index category, and 56.3% were working. Slightly more than 55% of women respondents were from communities with high media exposure and 51.5% were in union.

**Table 1:** Background characteristics of the study population (weighted)

Socio-demographic characteristics	Women		Men	
	N	Percentage	N	Percentage
**Age**				
Less than 25	6012	41.8	3633	37.1
25–34	4274	29.7	2331	23.8
35 and above	4085	28.4	3831	39.1
**Place of residence**				
Urban	6731	46.8	4157	42.4
Rural	7640	53.2	5638	57.6
**Region**				
Aire Métropolitaine de Port-au-Prince	3632	25.3	2308	23.6
Reste-Ouest	2285	15.9	1493	15.2
Grand Sud	1707	11.9	1323	13.5
Grand Nord	2778	19.3	1829	18.7
Artibonite/Centre	3008	20.9	2029	20.7
Grand’Anse/Nippes	959	6.7	812	8.3
**Education level**				
No education	1915	13.3	1445	14.8
Primary	4343	30.2	2877	29.4
Secondary	7068	49.2	4570	46.7
Higher	1045	7.3	902	9.2
**Religion**				
Christian	13,055	90.8	8015	81.8
Non-Christian	1316	9.2	1780	18.2
**Wealth Index**				
Poorest	2168	15.1	1706	17.4
Poorer	2428	16.9	1750	17.9
Middle	2772	19.3	2035	20.8
Richer	3396	23.6	2033	20.8
Richest	3607	25.1	2271	23.2
**Currently working**				
Yes	8091	56.3	7553	77.1
No	6280	43.7	2242	22.9
**Community media level**				
High	8148	56.7	5073	51.8
Low	6223	43.3	4722	48.2
**Marital status**				
Never in union	5823	40.5	4646	47.4
In union	7402	51.5	4541	46.4
Divorced/Separated/Widowed	1146	8.0	608	6.2
**Total**	**14,371**	**100.0**	**9795**	**100.0**

As for the men, 37.1% were young and 39.1% were 35 and above. Their mean age was 33.0 (SD ± 14.2). Further, 23.6% came from the “Aire Métropolitaine de Port-au-Prince” region and 57.6% resided in rural areas. Slightly more than 80% of them were Christians, 46.7% had secondary education level, 9.2% had higher education level, and 14.8% had no formal education. More than three quarters were working and more than a third were in the poor wealth index category. Just over half (51.8%) of men were from communities with high media exposure and 46.4% were in union.

### Bivariate analysis of the relationship between Tobacco use and community and individual characteristics

The prevalence of tobacco use among women was 1.7% (95% CI: 1.5–1.9). Noticeable differences were observed according to community and individual characteristics (Table 2). Tobacco use was commonest among 35 and above-year age-group (2.6%), followed by 25–34-year age-group (1.8%), and least among 15-24-year age-group (0.9%). Results also revealed that tobacco use was much higher in urban areas (2.1%) and in the “Aire Métropolitaine de Port-au-Prince” region (3.1%). This practice was most prevalent among women with no formal education (3.5%), and least prevalent among those with higher education level (1.2%). As expected, tobacco use prevalence was four times higher among non-Christians (5.1%) than Christians (1.3%). Likewise, tobacco use was most frequent among women from poorest households (2.2%), who were working (1.8%), and who were from communities with high media exposure (1.9%). In addition, it is found that 4.2% of divorced/separated/widowed women were using tobacco, while this proportion was 1.8% and 1.0% among those in union and who had been never married, respectively.

**Table 2:** Prevalence of tobacco use by individual and community characteristics (weighted)

Socio-demographic characteristics	Women		*P*-value (χ^2^)	Men		*P*-value (χ^2^)
	Yes (N/%)	No (N/%)		Yes (N/%)	No (N/%)	
**Age**			< 0.001			< 0.001
Less than 25	55 (0.9)	5957 (99.1)		166 (4.6)	3467 (95.4)	
25–34	77 (1.8)	4198 (98.2)		240 (10.3)	2091 (89.7)	
35 and above	107 (2.6)	3978 (97.4)		556 (14.5)	3275 (85.5)	
**Place of residence**			< 0.001			0.644
Urban	142 (2.1)	6588 (97.9)		415 (10.0)	3742 (90.0)	
Rural	96 (1.3)	7544 (98.7)		547 (9.7)	5091 (90.3)	
**Region**			< 0.001			< 0.01
Aire Métropolitaine de Port-au-Prince	111 (3.1)	3521 (96.9)		262 (11.3)	2047 (88.7)	
Reste-Ouest	32 (1.4)	2253 (98.6)		126 (8.4)	1367 (91.6)	
Grand Sud	15 (0.9)	1693 (99.1)		125 (9.4)	1198 (90.6)	
Grand Nord	23 (0.8)	2755 (99.2)		151 (8.3)	1677 (91.7)	
Artibonite/Centre	47 (1.6)	2961 (98.4)		223 (11.0)	1806 (89.0)	
Grand’Anse/Nippes	9 (0.9)	950 (99.1)		75 (9.2)	737 (90.8)	
**Education level**			< 0.001			< 0.001
No education	67 (3.5)	1848 (96.5)		281 (19.4)	1165 (80.6)	
Primary	64 (1.5)	4280 (98.5)		325 (11.3)	2553 (88.7)	
Secondary	95 (1.3)	6973 (98.7)		300 (6.6)	4270 (93.4)	
Higher	13 (1.2)	1032 (98.8)		57 (6.3)	845 (93.7)	
**Religion**			< 0.001			< 0.001
Christian	172 (1.3)	12,883 (98.7)		623 (7.8)	7392 (92.2)	
Non-Christian	67 (5.1)	1250 (94.9)		339 (19.0)	1441 (81.0)	
**Wealth Index**			0.067			< 0.001
Poorest	48 (2.2)	2119 (97.8)		239 (14.0)	1467 (86.0)	
Poorer	33 (1.4)	2396 (98.6)		191 (10.9)	1559 (89.1)	
Middle	42 (1.5)	2730 (98.5)		184 (9.0)	1851 (91.0)	
Richer	47 (1.4)	3349 (98.6)		177 (8.7)	1856 (91.3)	
Richest	69 (1.9)	3539 (98.1)		171 (7.5)	2100 (92.5)	
**Currently working**			0.187			< 0.001
Yes	144 (1.8)	7947 (98.2)		840 (11.1)	6713 (88.9)	
No	94 (1.5)	6186 (98.5)		122 (5.4)	2120 (94.6)	
**Community media levl**			< 0.05			< 0.01
High	151 (1.9)	7997 (98.1)		458 (9.0)	4615 (91.0)	
Low	87 (1.4)	6135 (98.6)		504 (10.7)	4218 (89.3)	
**Marital status**			< 0.001			< 0.001
Never in union	56 (1.0)	5767 (99.0)		208 (4.5)	4438 (95.5)	
In union	135 (1.8)	7267 (98.2)		605 (13.3)	3936 (86.7)	
Divorced/Separated/Widowed	48 (4.2)	1098 (95.8)		149 (24.5)	459 (75.5)	
**Total**	**239 (1.7)**	**14,132 (98.3)**		**962 (9.8)**	**8833 (90.2)**	

The prevalence of tobacco use in men was estimated at 9.8% (95% CI: 9.2–10.4), almost 6 times higher than in women. Furthermore, as with women, tobacco use was most prevalent among men who were in the 35 + age group (14.5%), from urban areas (10.0%), “Aire Métropolitaine de Port-au-Prince” (11.3%) and poorest households (14.0%), non-Christians (19.0%), with no formal education level (19.4%), with an income-generating activity (11.1%), and who were divorced/separated/widowed (24.5%). Unlike women, tobacco use was slightly more frequent among men from communities with low media exposure (10.7%) than those from communities with high media exposure (9.8%).

### Multilevel regression and factors associated with tobacco use

Table 3 displays the multi-level logistic regression for women. Results showed that women aged 25–34 years (aOR = 2.04; 95% CI: 1.33–3.14) and 35 and above (aOR = 2.73; 95% CI: 1.70–4.38) had higher odds of tobacco use compared to their counterparts aged less than 25 years. Respondents with no formal education were 2.3 times more likely to use tobacco (aOR = 2.29; 95% CI: 1.11–4.72) than those with higher level. Non-Christians (aOR = 3.79; 95% CI: 2.69–5.35) were found to have a higher probability of tobacco use. Similarly, the odds of tobacco use were 3.8 (aOR = 3.76; 95% CI: 1.80–7.84) times higher for women from poorest households compared to those from richest households. Having been divorced/separated/widowed was associated with higher risk (aOR = 2.36; 95% CI: 1.42–3.93) of tobacco use, while having an income-generating activity was associated with 31% lower risk (aOR = 0.69; 95% CI: 0.51–0.94) of tobacco use. Compared to women from the “Aire Métropolitaine de Port-au-Prince” region, those from Reste-Ouest (aOR = 0.41; 95% CI: 0.19–0.86), Grand Sud (aOR = 0.29; 95% CI: 0.13–0.65), Grand Nord (aOR = 0.25; 95% CI: 0.13–0.48), Artibonite/Centre (aOR = 0.35; 95% CI: 0.18–0.65), and Grand’Anse/Nippes (aOR = 0.26; 95% CI: 0.11–0.65) were less likely to use tobacco. Finally, women from communities with high media exposure had 1.8 greater odds (aOR = 1.77; 95% CI: 1.02–3.09) to use tobacco.

**Table 3:** Multilevel parameter estimates and adjusted odds of tobacco use (for women)

Socio-demographic characteristics	ICC = 27.81%	aOR (95% CI)	aOR (95% CI)	aOR (95% CI)
	**Model-0**	**Model-I**	**Model-II**	**Model-III**
Age				
25–34		2.05 (1.33–3.15)**		2.04 (1.33–3.14)**
35 and above		2.71 (1.69–4.35)***		2.73 (1.70–4.38)***
Ref = Less than 25				
**Education level**				
No education		2.25 (1.09–4.63)*		2.29 (1.11–4.72)*
Primary		1.20 (0.61–2.36)		1.24 (0.63–2.45)
Secondary		1.40 (0.75–2.60)		1.46 (0.79–2.71)
Ref = Higher				
**Religion**				
Non-Christian		4.12 (2.93–5.81)***		3.79 (2.69–5.35)***
Ref = Christian				
**Wealth Index**				
Poorest		0.99 (0.58–1.70)		3.76 (1.80–7.84)***
Poorer		0.60 (0.35–1.04)		1.92 (0.97–3.80)
Middle		0.61 (0.37–0.98)*		1.17 (0.69–1.98)
Richer		0.64 (0.42–0.97)*		0.80 (0.52–1.21)
Ref = Richest				
**Currently working**				
Yes		0.70 (0.52–0.96)*		0.69 (0.51–0.94)*
Ref = No				
**Marital status**				
In union		1.03 (0.67–1.58)		1.05 (0.69–1.61)
Divorced/Separated/Widowed		2.46 (1.47–4.09)***		2.36 (1.42–3.93)***
Ref = Never in union				
**Community media level**				
High			0.94 (0.56–1.57)	1.77 (1.02–3.09)*
Ref = Low				
**Place of residence**				
Urban			1.17 (0.62–2.19)	0.69 (0.36–1.33)
Ref = Rural				
**Region**				
Reste-Ouest			0.33 (0.15–0.73)**	0.41 (0.19–0.86)*
Grand Sud			0.21 (0.09–0.48)***	0.29 (0.13–0.65)**
Grand Nord			0.19 (0.09–0.38)***	0.25 (0.13–0.48)***
Artibonite/Centre			0.38 (0.20–0.74)**	0.35 (0.18–0.65)**
Grand’Anse/Nippes			0.23 (0.09–0.59)**	0.26 (0.11–0.65)**
Ref = Aire Métropolitaine de Port-au-Prince				
**Measure of variation**				
Variance	1.27 (0.74–1.79)	1.05 (0.59–1.51)	1.01 (0.55–1.46)	0.79 (0.40–1.19)
ICC (%)	27.81	24.22	23.36	19.41
PCV (%)	Reference	17.32	20.47	37.80
**Model fitness**				
Log-likelihood	− 1162.87	− 1088.45	− 1145.40	− 1065.56
AIC	2329.74	2206.90	2308.80	2175.12

ICC intra-class Correlation Coefficient, PCV proportional change in variance, AIC Akaike Information Criterion

*p < 0.05, **p < 0.01, ***p < 0.001

Results also indicated that men aged 35 and above (aOR = 1.58; 95% CI: 1.18–2.11), with no formal education (aOR = 2.45; 95% CI: 1.69–3.56), Non-Christians (aOR = 2.89; 95% CI: 2.46–3.42), from poorest households (aOR = 2.25; 95% CI: 1.55–3.28), divorced/separated/widowed (aOR = 4.27; 95% CI: 3.14–5.79), from rural areas (Ref) and “Aire Métropolitaine de Port-au-Prince” region (Ref) had a higher likelihood of tobacco use (Table 4).

**Table 4:** Multilevel parameter estimates and adjusted odds of tobacco use (for men)

Socio-demographic characteristics	ICC = 9.68%	aOR (95% CI)	aOR (95% CI)	aOR (95% CI)
	**Model-0**	**Model-I**	**Model-II**	**Model-III**
Age				
25–34		1.44 (1.09–1.89)**		1.43 (1.09–1.88)**
35 and above		1.57 (1.18–2.11)**		1.58 (1.18–2.11)**
Ref = Less than 25				
**Education level**				
No education		2.33 (1.61–3.38)***		2.45 (1.69–3.56)***
Primary		1.71 (1.21–2.42)**		1.78 (1.25–2.52)**
Secondary		1.17 (0.85–1.62)		1.21 (0.88–1.68)
Ref = Higher				
**Religion**				
Non-Christian		2.90 (2.46–3.41)***		2.89 (2.46–3.42)***
Ref = Christian				
**Wealth Index**				
Poorest		1.14 (0.85–1.53)		2.25 (1.55–3.28)***
Poorer		0.94 (0.70–1.26)		1.75 (1.22–2.49)**
Middle		0.81 (0.62–1.07)		1.23 (0.90–1.66)
Richer		0.88 (0.69–1.13)		1.03 (0.80–1.33)
Ref = Richest				
**Currently working**				
Yes		1.07 (0.84–1.36)		1.11 (0.88–1.41)
Ref = No				
**Marital status**				
In union		2.15 (1.67–2.77)***		2.16 (1.68–2.77)***
Divorced/Separated/Widowed		4.37 (3.21–5.94)***		4.27 (3.14–5.79)***
Ref = Never in union				
**Community media level**				
High			0.75 (0.59–0.95)*	1.16 (0.90–1.49)
Ref = Low				
**Place of residence**				
Urban			1.02 (0.77–1.35)	0.62 (0.46–0.83)**
Ref = Rural				
**Region**				
Reste-Ouest			0.57 (0.38–0.84)**	0.52 (0.35–0.77)***
Grand Sud			0.67 (0.46–0.98)*	0.69 (0.48–1.01)
Grand Nord			0.61 (0.44–0.86)**	0.63 (0.46–0.88)**
Artibonite/Centre			0.76 (0.54–1.06)	0.60 (0.43–0.84)**
Grand’Anse/Nippes			0.65 (0.44–0.98)*	0.59 (0.40–0.89)*
Ref = Aire Métropolitaine de Port-au-Prince				
**Measure of variation**				
Variance	0.35 (0.23–0.47)	0.32 (0.20–0.43)	0.31 (0.20–0.42)	0.24 (0.13–0.34)
ICC (%)	9.68	8.76	8.60	6.71
PCV (%)	Reference	8.57	11.43	31.43
**Model fitness**				
Log-likelihood	− 3095.40	− 2804.25	− 3087.09	− 2778.46
AIC	6194.80	5638.49	6192.18	5600.91

ICC intra-class Correlation Coefficient, PCV proportional change in variance, AIC Akaike Information Criterion

*p < 0.05, **p < 0.01, ***p < 0.001

#### Random effects analysis (for women)

The ICC in the null model (ICC = 27.8%) showed that the odds of tobacco use varied across clusters (σ^2^ = 1.27; 95% CI: 0.74–1.79). Besides, the PCV in the final model (Model-III) revealed that 37.8% of the variability in tobacco use was explained by both individual and community-level characteristics (Table 3).

#### Random effects analysis (for men)

The null model indicated that the ICC of 9.7% total variability for tobacco use was due to changes between clusters, and the remaining unexplained was attributable to within-cluster variability. Additionally, the PCV of the multilevel model showed that 31.4% of the variability in tobacco use was explained by the full model (Table 4).

## Discussion

As far as we know, this study is the first to provide evidence about tobacco use in Haiti. To ensure optimum generalizability of our findings, we used the 2016/17 HDHS. Specifically, this study determined the prevalence and individual and community-level factors associated with tobacco use in Haiti for women and men separately. Results indicated that tobacco use prevalence was 6 times higher among men (9.8%) than women (1.7%). Findings also revealed that age, education level, religion, wealth index, working status, marital status, community level media exposure, place of residence and region significantly influenced tobacco use.

The prevalence of tobacco use was found to be markedly higher among men than women in Haiti. This finding is in concordance with several previous studies [26, 47, 55]. There might be a reason for this because women have a higher risk of developing tobacco-related morbidity and mortality than men [56]. Additionally, this could be related to gender norms in Haitian society [57]. In many families/regions, a woman who smokes cigarettes or uses any other type of tobacco is seen as a “deviant”. Consequently, she may be stigmatized and rejected by her family/community [58].

In this study, respondents aged less than 25 years old had a lower risk of using tobacco, which is supported by studies conducted in Rwanda [24] and Ethiopia [59]. Youth are prohibited from using this substance in Haiti [60]. Further, this lower risk among youth could also be explained by the fact that individuals progressively start smoking as they get older [55].

Respondents with no formal education faced a greater risk of tobacco use than those with higher education level. Congruent with previous studies [31, 32], this finding confirms that educational attainment acts as a safety net against tobacco use. Arguably, respondents with higher education levels may be better informed about the harmful effects of tobacco use [32].

Our results highlighted that being from the “Aire Métropolitaine de Port-au-Prince” region (which is the capital) increased the likelihood of tobacco use. This finding is consistent with prior studies [24, 43, 44, 61], which demonstrated that regional variation is an important predictor of tobacco use. This might be greatly due to a better availability and access to tobacco in the “Aire Métropolitaine de Port-au-Prince” region.

Place of residence is another important predictor of tobacco use. Men from rural areas had higher odds of tobacco use than their urban counterparts. This result corroborates previous studies [62, 63]. This probably reflects different factors. First, tobacco products are accessible at low prices in rural areas where tobacco is still produced. Second, men from rural areas are less educated and less exposed to health-related information than those from urban areas [42].

Marital status was also a determinant of tobacco use. Respondents who were divorced/separated/widowed had significantly higher odds of tobacco use as compared to those who were never in union. Earlier studies have reported similar findings [43, 45, 64]. In the wake of a divorce, separation, or the death of a spouse, many people turn to alcohol and tobacco to cope with their loss [65]. Furthermore, our data indicated that participants (women or men) who were never in a union were younger than those who were divorced/separated/widowed, which might decrease their odds of tobacco use.

Participants from poor households had higher risks of tobacco use. This aligns with past studies conducted in India [66] and Iran [67] while this result is in contrast to the study by Kulkarni et al. in southern India [68]. People struggling with adverse conditions are generally affected physically and psychologically, and are also vulnerable to natural and unnatural stresses [69]. They may acquire substances such as tobacco to suppress or avoid negative feelings and to deal with the tensions of material deprivation [43]. However, note that there is a bilateral relationship between being poor and tobacco use. Numerous studies have indicated that tobacco abuse can result in a significant decrease in household income, thus contributing to the harshness of poor living conditions [43, 70]. Therefore, this justifies the need for increasing taxes on tobacco products to reduce the access to tobacco products. As argued by Bosdriesz et al. [71], when taxes are lower, tobacco becomes more affordable for low socioeconomic status groups.

The present study also included religion as a predictive variable. The results of prior studies indicated that being a Christian reduced the risk of tobacco use [72–74]. The Haitian Christian community abstains from consumption of substances because they believe Scripture condemns them. Travis Hirschi argued that religious training prevents people from engaging in “deviant” behavior by promoting moral values and conformity [75, 76]. Furthermore, it is not uncommon for congregants to be sanctioned by Haitian church leaders if they discover that they are using tobacco. The Haitian Christian community and particularly church leaders stigmatized individuals who use tobacco. Therefore, the church community is seen as being very instrumental to influencing the congregants’ behaviors [77].

The study further revealed that tobacco use was significantly associated with working status. The odds of tobacco use were higher among women who were not working. The result contradicts previous studies [32, 55]. This difference could be attributed to the vulnerable economic conditions of women who were not working. Generally, poor people have less control to deal with the management of stress from their economic situations [55].

Similarly, being from communities with high media exposure was associated with higher risks of tobacco use. Previously available literature also noticed that individuals with high exposure to mass media might have an elevated risk of tobacco use [33, 44]. Nowadays, the mass media are a powerful means for disseminating information and reaching a large audience [78]. The last two decades have seen cigarettes promoted through mass media in Haiti. In the advertisements, it was presented in an overwhelmingly positive light. A growing body of research has shown that this marketing strategy may be powerful enough to modify individual’s attitudes about this substance [44, 79, 80].

### Policy implication of findings

Given that tobacco use prevalence is higher among respondents aged 35 and above, the Haitian Ministry of Public Health and Population should develop targeted and appropriate evidence-based interventions for reducing tobacco use among this population. Although the prevalence of tobacco use in young people is low, it increases considerably with age. This implies that there is a window of opportunity to intervene before they start smoking. Previous studies already demonstrated that early age of onset of tobacco use is related to continued use later in life [81, 82]. Community awareness programs on tobacco use impacts on youth, women and other vulnerable groups should be organized frequently to educate community members on adverse effects of tobacco on body and brain. Adult and elderly men who use tobacco should be specifically targeted by public policies aimed at reducing tobacco-related health issues both for themselves and other people they may expose to passive smoking. Community opinion leaders and associations should be involved in the health program development and implementation to create ownership. Smokeless tobacco products should be targeted, particularly in rural areas where culturally pipe full of tobacco, chews tobacco, and nasal snuff tobacco are used.

Advertisement agencies should be controlled and used to create healthier consumption behaviors. Political decision-makers must enforce official rules, such as restricting tobacco products marketing to people under 18 years old (minors), particularly in places like schools and others frequented by young people. They must create new rules prohibiting smoking in public places, rooms where there are children or other vulnerable groups, and preventing mass media advertising that encouraging young people to use tobacco. Furthermore, continuous health education using the various media (including social media) must be encouraged to create awareness about harmful consequences of tobacco use. Deterrent taxation policy can help the government to both reduce the access to tobacco products and fund such sensibilization campaign. The formation of Community Youth volunteer groups should be encouraged where ex-smokers share their lived experiences. Religious leaders too can help health promoters to influence behaviors and prevent tobacco addiction.

Specific interventions must be led in the “Aire Métropolitaine de Port-au-Prince” region, where the prevalence of tobacco use is the highest. Tobacco industries might be profitable for the private sector; the wealth it creates cannot exceed its health costs for a country. There is need to invest in research on tobacco addiction and related mental health effects to better inform targeted policies.

### Strengths and limitations of the study

The large sample size used in this study improved its statistical power. Weight application and analysis using a complex samples plan accounted for sampling biases and sampling designs, thereby producing unbiased national estimates. However, the cross–sectional nature of this study limits causality. In addition, the recall biases due to self-reporting of health behavior outcomes associated with DHS surveys could have influenced the study findings. Another limitation of this study is the use of secondary data which limits the selection and inclusion of other important variables that could have influenced the study findings. For instance, there was no information on the quantity of tobacco consumed, the use of e-cigarettes or illegal smoked substance such as bidis, marijuana, and cannabis.

## Conclusion

Findings from this study showed that the prevalence of tobacco use in Haiti was 9.8% among men and 1.7% among women. Our results also found that age, educational level, religion, wealth index, working status, marital status, mass media exposure, place of residence, and region were significantly influencing tobacco use in Haiti. The study revealed that prevalence of tobacco use was lower among women and young people. This lower prevalence, associated with low income, opens a window of opportunity to design appropriate rules and implement policies dissuading young people from starting to smoke, as tobacco use increases with the age. Adult male smokers should be targeted by appropriate policy to reduce the different health burdens associated with this substance, both for the smokers and other people they may expose to passive smoking.

Future studies should examine the determinants of tobacco use among the whole population as well as spatial analysis to identify areas with high tobacco use. Finally, qualitative studies should be conducted to better explore the intensity of tobacco use in Haiti, particularly among vulnerable groups such as childbearing women and elderly. Smokeless tobacco and other illegal smoked substance need to be studied.

## Data Availability

The data used in this study is publicly available at: https://dhsprogram.com/data/availabledatasets.cfm.
